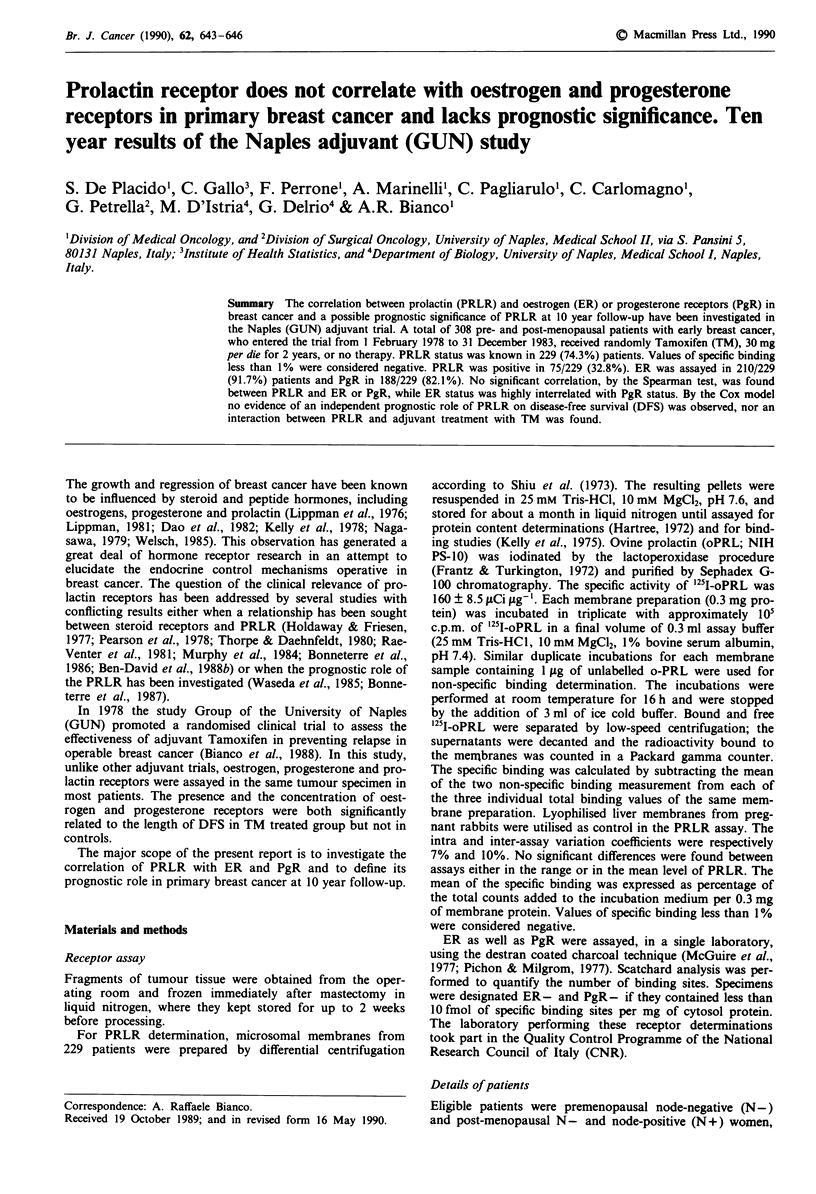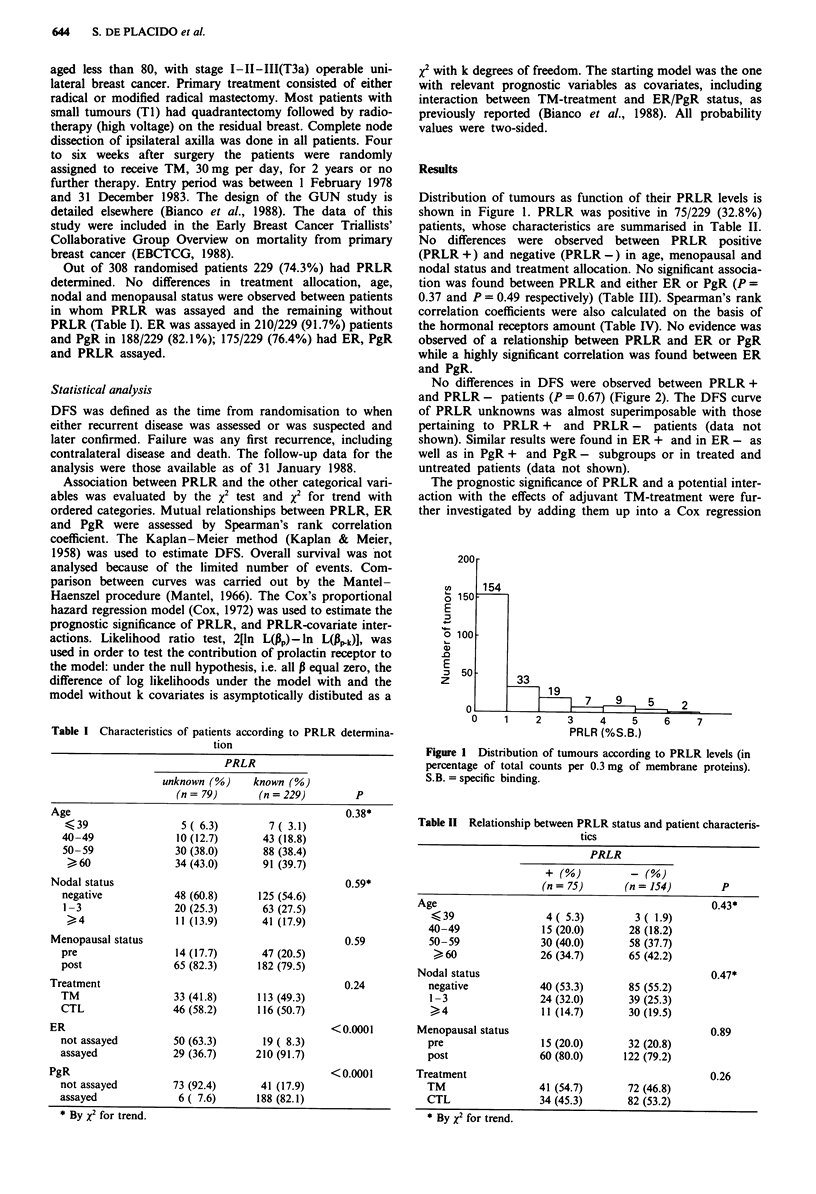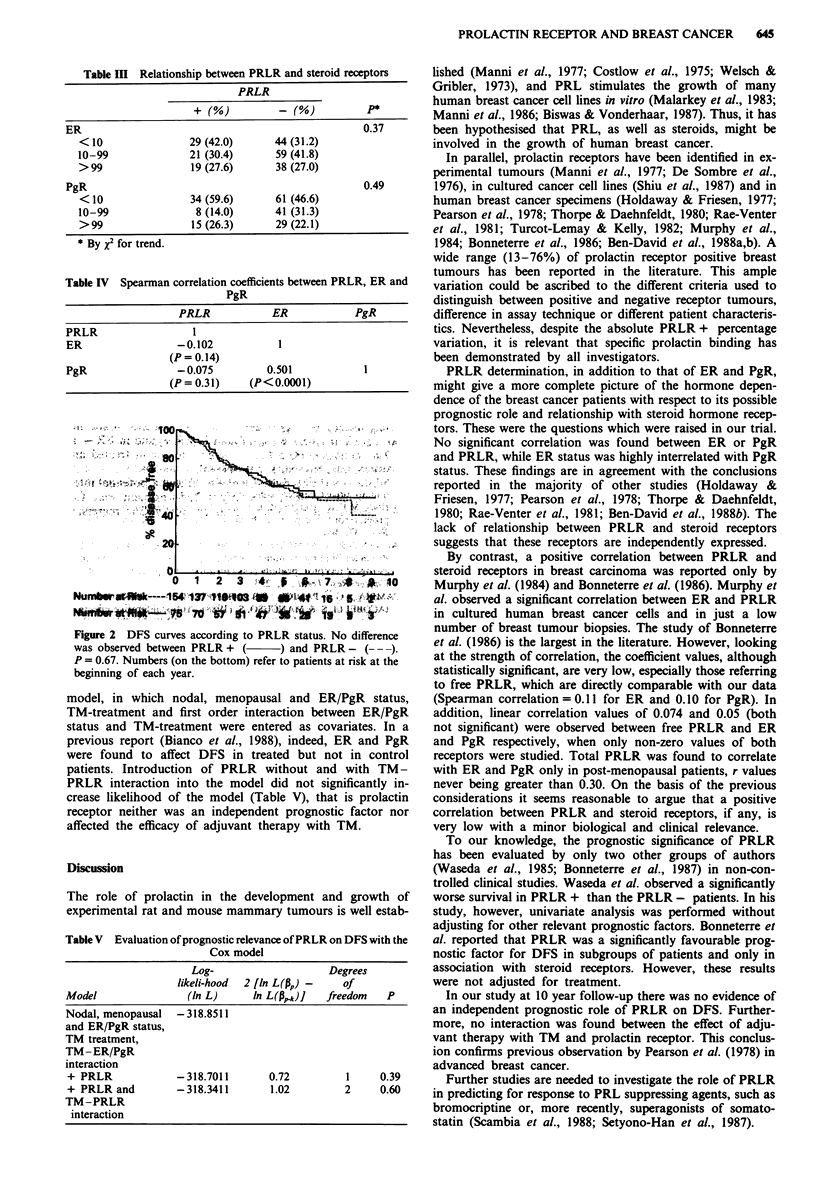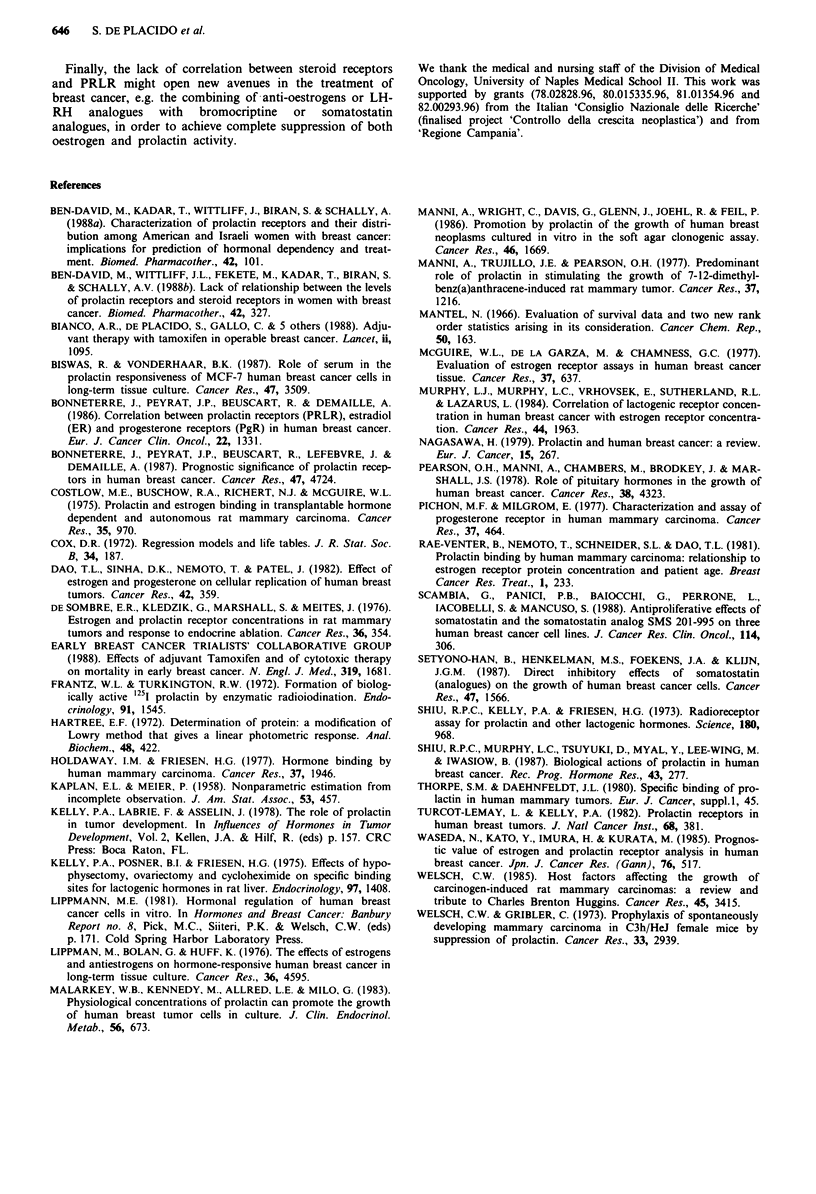# Prolactin receptor does not correlate with oestrogen and progesterone receptors in primary breast cancer and lacks prognostic significance. Ten year results of the Naples adjuvant (GUN) study.

**DOI:** 10.1038/bjc.1990.346

**Published:** 1990-10

**Authors:** S. De Placido, C. Gallo, F. Perrone, A. Marinelli, C. Pagliarulo, C. Carlomagno, G. Petrella, M. D'Istria, G. Delrio, A. R. Bianco

**Affiliations:** Division of Medical Oncology, University of Naples, Medical School II, Italy.

## Abstract

The correlation between prolactin (PRLR) and oestrogen (ER) or progesterone receptors (PgR) in breast cancer and a possible prognostic significance of PRLR at 10 year follow-up have been investigated in the Naples (GUN) adjuvant trial. A total of 308 pre- and post-menopausal patients with early breast cancer, who entered the trial from 1 February 1978 to 31 December 1983, received randomly Tamoxifen (TM), 30 mg per die for 2 years, or no therapy. PRLR status was known in 229 (74.3%) patients. Values of specific binding less than 1% were considered negative. PRLR was positive in 75/229 (32.8%). ER was assayed in 210/229 (91.7%) patients and PgR in 188/229 (82.1%). No significant correlation, by the Spearman test, was found between PRLR and ER or PgR, while ER status was highly interrelated with PgR status. By the Cox model no evidence of an independent prognostic role of PRLR on disease-free survival (DFS) was observed, nor an interaction between PRLR and adjuvant treatment with TM was found.


Br. J. Cancer (1990), 62, 643–646 © Macmillan Press Ltd., 1990

### Prolactin receptor does not correlate with oestrogen and progesterone receptors in primary breast cancer and lacks prognostic significance. Ten year results of the Naples adjuvant (GUN) study

S. De Placido[1], C. Gallo[3], F. Perrone[1], A. Marinelli[1], C. Pagliarulo[1], C. Carlomagno[1], G. Petrella[2], M. D'Istria[4], G. Delrio[4] & A.R. Bianco[1]

[1]*Division of Medical Oncology, and* [2]*Division of Surgical Oncology, University of Naples, Medical School II, via S. Pansini 5, 80131 Naples, Italy;* [3]*Institute of Health Statistics, and* [4]*Department of Biology, University of Naples, Medical School I, Naples, Italy.*

**Summary** The correlation between prolactin (PRLR) and oestrogen (ER) or progesterone receptors (PgR) in breast cancer and a possible prognostic significance of PRLR at 10 year follow-up have been investigated in the Naples (GUN) adjuvant trial. A total of 308 pre- and post-menopausal patients with early breast cancer, who entered the trial from 1 February 1978 to 31 December 1983, received randomly Tamoxifen (TM), 30 mg *per die* for 2 years, or no therapy. PRLR status was known in 229 (74.3%) patients. Values of specific binding less than 1% were considered negative. PRLR was positive in 75/229 (32.8%). ER was assayed in 210/229 (91.7%) patients and PgR in 188/229 (82.1%). No significant correlation, by the Spearman test, was found between PRLR and ER or PgR, while ER status was highly interrelated with PgR status. By the Cox model no evidence of an independent prognostic role of PRLR on disease-free survival (DFS) was observed, nor an interaction between PRLR and adjuvant treatment with TM was found.

The growth and regression of breast cancer have been known to be influenced by steroid and peptide hormones, including oestrogens, progesterone and prolactin (Lippman *et al.*, 1976; Lippman, 1981; Dao *et al.*, 1982; Kelly *et al.*, 1978; Nagasawa, 1979; Welsch, 1985). This observation has generated a great deal of hormone receptor research in an attempt to elucidate the endocrine control mechanisms operative in breast cancer. The question of the clinical relevance of prolactin receptors has been addressed by several studies with conflicting results either when a relationship has been sought between steroid receptors and PRLR (Holdaway & Friesen, 1977; Pearson *et al.*, 1978; Thorpe & Daehnfeldt, 1980; Rae-Venter *et al.*, 1981; Murphy *et al.*, 1984; Bonneterre *et al.*, 1986; Ben-David *et al.*, 1988*b*) or when the prognostic role of the PRLR has been investigated (Waseda *et al.*, 1985; Bonneterre *et al.*, 1987).

In 1978 the study Group of the University of Naples (GUN) promoted a randomised clinical trial to assess the effectiveness of adjuvant Tamoxifen in preventing relapse in operable breast cancer (Bianco *et al.*, 1988). In this study, unlike other adjuvant trials, oestrogen, progesterone and prolactin receptors were assayed in the same tumour specimen in most patients. The presence and the concentration of oestrogen and progesterone receptors were both significantly related to the length of DFS in TM treated group but not in controls.

The major scope of the present report is to investigate the correlation of PRLR with ER and PgR and to define its prognostic role in primary breast cancer at 10 year follow-up.

### Materials and methods

### *Receptor assay*

Fragments of tumour tissue were obtained from the operating room and frozen immediately after mastectomy in liquid nitrogen, where they kept stored for up to 2 weeks before processing.

For PRLR determination, microsomal membranes from 229 patients were prepared by differential centrifugation according to Shiu *et al.* (1973). The resulting pellets were resuspended in 25 mM Tris-HCl, 10 mM $MgCl_2$, pH 7.6, and stored for about a month in liquid nitrogen until assayed for protein content determinations (Hartree, 1972) and for binding studies (Kelly *et al.*, 1975). Ovine prolactin (oPRL; NIH PS-10) was iodinated by the lactoperoxidase procedure (Frantz & Turkington, 1972) and purified by Sephadex G-100 chromatography. The specific activity of $^{125}$I-oPRL was $160 \pm 8.5\ \mu Ci\ \mu g^{-1}$. Each membrane preparation (0.3 mg protein) was incubated in triplicate with approximately $10^5$ c.p.m. of $^{125}$I-oPRL in a final volume of 0.3 ml assay buffer (25 mM Tris-HCl, 10 mM $MgCl_2$, 1% bovine serum albumin, pH 7.4). Similar duplicate incubations for each membrane sample containing 1 μg of unlabelled o-PRL were used for non-specific binding determination. The incubations were performed at room temperature for 16 h and were stopped by the addition of 3 ml of ice cold buffer. Bound and free $^{125}$I-oPRL were separated by low-speed centrifugation; the supernatants were decanted and the radioactivity bound to the membranes was counted in a Packard gamma counter. The specific binding was calculated by subtracting the mean of the two non-specific binding measurement from each of the three individual total binding values of the same membrane preparation. Lyophilised liver membranes from pregnant rabbits were utilised as control in the PRLR assay. The intra and inter-assay variation coefficients were respectively 7% and 10%. No significant differences were found between assays either in the range or in the mean level of PRLR. The mean of the specific binding was expressed as percentage of the total counts added to the incubation medium per 0.3 mg of membrane protein. Values of specific binding less than 1% were considered negative.

ER as well as PgR were assayed, in a single laboratory, using the destran coated charcoal technique (McGuire *et al.*, 1977; Pichon & Milgrom, 1977). Scatchard analysis was performed to quantify the number of binding sites. Specimens were designated ER– and PgR– if they contained less than 10 fmol of specific binding sites per mg of cytosol protein. The laboratory performing these receptor determinations took part in the Quality Control Programme of the National Research Council of Italy (CNR).

### *Details of patients*

Eligible patients were premenopausal node-negative (N–) and post-menopausal N– and node-positive (N+) women,

Correspondence: A. Raffaele Bianco.
Received 19 October 1989; and in revised form 16 May 1990.

aged less than 80, with stage I–II–III(T3a) operable unilateral breast cancer. Primary treatment consisted of either radical or modified radical mastectomy. Most patients with small tumours (T1) had quadrantectomy followed by radiotherapy (high voltage) on the residual breast. Complete node dissection of ipsilateral axilla was done in all patients. Four to six weeks after surgery the patients were randomly assigned to receive TM, 30 mg per day, for 2 years or no further therapy. Entry period was between 1 February 1978 and 31 December 1983. The design of the GUN study is detailed elsewhere (Bianco et al., 1988). The data of this study were included in the Early Breast Cancer Triallists' Collaborative Group Overview on mortality from primary breast cancer (EBCTCG, 1988).

Out of 308 randomised patients 229 (74.3%) had PRLR determined. No differences in treatment allocation, age, nodal and menopausal status were observed between patients in whom PRLR was assayed and the remaining without PRLR (Table I). ER was assayed in 210/229 (91.7%) patients and PgR in 188/229 (82.1%); 175/229 (76.4%) had ER, PgR and PRLR assayed.

### *Statistical analysis*

DFS was defined as the time from randomisation to when either recurrent disease was assessed or was suspected and later confirmed. Failure was any first recurrence, including contralateral disease and death. The follow-up data for the analysis were those available as of 31 January 1988.

Association between PRLR and the other categorical variables was evaluated by the $\chi^2$ test and $\chi^2$ for trend with ordered categories. Mutual relationships between PRLR, ER and PgR were assessed by Spearman's rank correlation coefficient. The Kaplan–Meier method (Kaplan & Meier, 1958) was used to estimate DFS. Overall survival was not analysed because of the limited number of events. Comparison between curves was carried out by the Mantel–Haenszel procedure (Mantel, 1966). The Cox's proportional hazard regression model (Cox, 1972) was used to estimate the prognostic significance of PRLR, and PRLR-covariate interactions. Likelihood ratio test, $2[\ln L(\beta_p) - \ln L(\beta_{p-k})]$, was used in order to test the contribution of prolactin receptor to the model: under the null hypothesis, i.e. all $\beta$ equal zero, the difference of log likelihoods under the model with and the model without k covariates is asymptotically distibuted as a $\chi^2$ with k degrees of freedom. The starting model was the one with relevant prognostic variables as covariates, including interaction between TM-treatment and ER/PgR status, as previously reported (Bianco et al., 1988). All probability values were two-sided.

**Table I** Characteristics of patients according to PRLR determination

| | PRLR unknown (%) (n = 79) | PRLR known (%) (n = 229) | P |
|---|---|---|---|
| Age | | | 0.38* |
| ⩽39 | 5 ( 6.3) | 7 ( 3.1) | |
| 40–49 | 10 (12.7) | 43 (18.8) | |
| 50–59 | 30 (38.0) | 88 (38.4) | |
| ⩾60 | 34 (43.0) | 91 (39.7) | |
| Nodal status | | | 0.59* |
| negative | 48 (60.8) | 125 (54.6) | |
| 1–3 | 20 (25.3) | 63 (27.5) | |
| ⩾4 | 11 (13.9) | 41 (17.9) | |
| Menopausal status | | | 0.59 |
| pre | 14 (17.7) | 47 (20.5) | |
| post | 65 (82.3) | 182 (79.5) | |
| Treatment | | | 0.24 |
| TM | 33 (41.8) | 113 (49.3) | |
| CTL | 46 (58.2) | 116 (50.7) | |
| ER | | | <0.0001 |
| not assayed | 50 (63.3) | 19 ( 8.3) | |
| assayed | 29 (36.7) | 210 (91.7) | |
| PgR | | | <0.0001 |
| not assayed | 73 (92.4) | 41 (17.9) | |
| assayed | 6 ( 7.6) | 188 (82.1) | |

* By $\chi^2$ for trend.

### Results

Distribution of tumours as function of their PRLR levels is shown in Figure 1. PRLR was positive in 75/229 (32.8%) patients, whose characteristics are summarised in Table II. No differences were observed between PRLR positive (PRLR +) and negative (PRLR −) in age, menopausal and nodal status and treatment allocation. No significant association was found between PRLR and either ER or PgR ($P = 0.37$ and $P = 0.49$ respectively) (Table III). Spearman's rank correlation coefficients were also calculated on the basis of the hormonal receptors amount (Table IV). No evidence was observed of a relationship between PRLR and ER or PgR while a highly significant correlation was found between ER and PgR.

No differences in DFS were observed between PRLR + and PRLR − patients ($P = 0.67$) (Figure 2). The DFS curve of PRLR unknowns was almost superimposable with those pertaining to PRLR + and PRLR − patients (data not shown). Similar results were found in ER + and in ER − as well as in PgR + and PgR − subgroups or in treated and untreated patients (data not shown).

The prognostic significance of PRLR and a potential interaction with the effects of adjuvant TM-treatment were further investigated by adding them up into a Cox regression

**Figure 1** Distribution of tumours according to PRLR levels (in percentage of total counts per 0.3 mg of membrane proteins). S.B. = specific binding.

**Table II** Relationship between PRLR status and patient characteristics

| | PRLR + (%) (n = 75) | PRLR − (%) (n = 154) | P |
|---|---|---|---|
| Age | | | 0.43* |
| ⩽39 | 4 ( 5.3) | 3 ( 1.9) | |
| 40–49 | 15 (20.0) | 28 (18.2) | |
| 50–59 | 30 (40.0) | 58 (37.7) | |
| ⩾60 | 26 (34.7) | 65 (42.2) | |
| Nodal status | | | 0.47* |
| negative | 40 (53.3) | 85 (55.2) | |
| 1–3 | 24 (32.0) | 39 (25.3) | |
| ⩾4 | 11 (14.7) | 30 (19.5) | |
| Menopausal status | | | 0.89 |
| pre | 15 (20.0) | 32 (20.8) | |
| post | 60 (80.0) | 122 (79.2) | |
| Treatment | | | 0.26 |
| TM | 41 (54.7) | 72 (46.8) | |
| CTL | 34 (45.3) | 82 (53.2) | |

* By $\chi^2$ for trend.

Table III Relationship between PRLR and steroid receptors

| | PRLR + (%) | PRLR − (%) | P* |
|---|---|---|---|
| ER | | | 0.37 |
| <10 | 29 (42.0) | 44 (31.2) | |
| 10–99 | 21 (30.4) | 59 (41.8) | |
| >99 | 19 (27.6) | 38 (27.0) | |
| PgR | | | 0.49 |
| <10 | 34 (59.6) | 61 (46.6) | |
| 10–99 | 8 (14.0) | 41 (31.3) | |
| >99 | 15 (26.3) | 29 (22.1) | |

* By $\chi^2$ for trend.

Table IV Spearman correlation coefficients between PRLR, ER and PgR

| | PRLR | ER | PgR |
|---|---|---|---|
| PRLR | 1 | | |
| ER | −0.102 ($P=0.14$) | 1 | |
| PgR | −0.075 ($P=0.31$) | 0.501 ($P<0.0001$) | 1 |

**Figure 2** DFS curves according to PRLR status. No difference was observed between PRLR + (———) and PRLR − (- - -). $P = 0.67$. Numbers (on the bottom) refer to patients at risk at the beginning of each year.

model, in which nodal, menopausal and ER/PgR status, TM-treatment and first order interaction between ER/PgR status and TM-treatment were entered as covariates. In a previous report (Bianco *et al.*, 1988), indeed, ER and PgR were found to affect DFS in treated but not in control patients. Introduction of PRLR without and with TM−PRLR interaction into the model did not significantly increase likelihood of the model (Table V), that is prolactin receptor neither was an independent prognostic factor nor affected the efficacy of adjuvant therapy with TM.

### Discussion

The role of prolactin in the development and growth of experimental rat and mouse mammary tumours is well established (Manni *et al.*, 1977; Costlow *et al.*, 1975; Welsch & Gribler, 1973), and PRL stimulates the growth of many human breast cancer cell lines *in vitro* (Malarkey *et al.*, 1983; Manni *et al.*, 1986; Biswas & Vonderhaar, 1987). Thus, it has been hypothesised that PRL, as well as steroids, might be involved in the growth of human breast cancer.

Table V Evaluation of prognostic relevance of PRLR on DFS with the Cox model

| Model | Log-likeli-hood (ln L) | $2 [\ln L(\beta_p) - \ln L(\beta_{p-k})]$ | Degrees of freedom | P |
|---|---|---|---|---|
| Nodal, menopausal and ER/PgR status, TM treatment, TM−ER/PgR interaction | −318.8511 | | | |
| + PRLR | −318.7011 | 0.72 | 1 | 0.39 |
| + PRLR and TM−PRLR interaction | −318.3411 | 1.02 | 2 | 0.60 |

In parallel, prolactin receptors have been identified in experimental tumours (Manni *et al.*, 1977; De Sombre *et al.*, 1976), in cultured cancer cell lines (Shiu *et al.*, 1987) and in human breast cancer specimens (Holdaway & Friesen, 1977; Pearson *et al.*, 1978; Thorpe & Daehnfeldt, 1980; Rae-Venter *et al.*, 1981; Turcot-Lemay & Kelly, 1982; Murphy *et al.*, 1984; Bonneterre *et al.*, 1986; Ben-David *et al.*, 1988a,b). A wide range (13–76%) of prolactin receptor positive breast tumours has been reported in the literature. This ample variation could be ascribed to the different criteria used to distinguish between positive and negative receptor tumours, difference in assay technique or different patient characteristics. Nevertheless, despite the absolute PRLR+ percentage variation, it is relevant that specific prolactin binding has been demonstrated by all investigators.

PRLR determination, in addition to that of ER and PgR, might give a more complete picture of the hormone dependence of the breast cancer patients with respect to its possible prognostic role and relationship with steroid hormone receptors. These were the questions which were raised in our trial. No significant correlation was found between ER or PgR and PRLR, while ER status was highly interrelated with PgR status. These findings are in agreement with the conclusions reported in the majority of other studies (Holdaway & Friesen, 1977; Pearson *et al.*, 1978; Thorpe & Daehnfeldt, 1980; Rae-Venter *et al.*, 1981; Ben-David *et al.*, 1988*b*). The lack of relationship between PRLR and steroid receptors suggests that these receptors are independently expressed.

By contrast, a positive correlation between PRLR and steroid receptors in breast carcinoma was reported only by Murphy *et al.* (1984) and Bonneterre *et al.* (1986). Murphy *et al.* observed a significant correlation between ER and PRLR in cultured human breast cancer cells and in just a low number of breast tumour biopsies. The study of Bonneterre *et al.* (1986) is the largest in the literature. However, looking at the strength of correlation, the coefficient values, although statistically significant, are very low, especially those referring to free PRLR, which are directly comparable with our data (Spearman correlation = 0.11 for ER and 0.10 for PgR). In addition, linear correlation values of 0.074 and 0.05 (both not significant) were observed between free PRLR and ER and PgR respectively, when only non-zero values of both receptors were studied. Total PRLR was found to correlate with ER and PgR only in post-menopausal patients, *r* values never being greater than 0.30. On the basis of the previous considerations it seems reasonable to argue that a positive correlation between PRLR and steroid receptors, if any, is very low with a minor biological and clinical relevance.

To our knowledge, the prognostic significance of PRLR has been evaluated by only two other groups of authors (Waseda *et al.*, 1985; Bonneterre *et al.*, 1987) in non-controlled clinical studies. Waseda *et al.* observed a significantly worse survival in PRLR+ than the PRLR− patients. In his study, however, univariate analysis was performed without adjusting for other relevant prognostic factors. Bonneterre *et al.* reported that PRLR was a significantly favourable prognostic factor for DFS in subgroups of patients and only in association with steroid receptors. However, these results were not adjusted for treatment.

In our study at 10 year follow-up there was no evidence of an independent prognostic role of PRLR on DFS. Furthermore, no interaction was found between the effect of adjuvant therapy with TM and prolactin receptor. This conclusion confirms previous observation by Pearson *et al.* (1978) in advanced breast cancer.

Further studies are needed to investigate the role of PRLR in predicting for response to PRL suppressing agents, such as bromocriptine or, more recently, superagonists of somatostatin (Scambia *et al.*, 1988; Setyono-Han *et al.*, 1987).

Finally, the lack of correlation between steroid receptors and PRLR might open new avenues in the treatment of breast cancer, e.g. the combining of anti-oestrogens or LH-RH analogues with bromocriptine or somatostatin analogues, in order to achieve complete suppression of both oestrogen and prolactin activity.

We thank the medical and nursing staff of the Division of Medical Oncology, University of Naples Medical School II. This work was supported by grants (78.02828.96, 80.015335.96, 81.01354.96 and 82.00293.96) from the Italian 'Consiglio Nazionale delle Ricerche' (finalised project 'Controllo della crescita neoplastica') and from 'Regione Campania'.

### References

BEN-DAVID, M., KADAR, T., WITTLIFF, J., BIRAN, S. & SCHALLY, A. (1988*a*). Characterization of prolactin receptors and their distribution among American and Israeli women with breast cancer: implications for prediction of hormonal dependency and treatment. *Biomed. Pharmacother.*, **42**, 101.

BEN-DAVID, M., WITTLIFF, J.L., FEKETE, M., KADAR, T., BIRAN, S. & SCHALLY, A.V. (1988*b*). Lack of relationship between the levels of prolactin receptors and steroid receptors in women with breast cancer. *Biomed. Pharmacother.*, **42**, 327.

BIANCO, A.R., DE PLACIDO, S., GALLO, C. & 5 others (1988). Adjuvant therapy with tamoxifen in operable breast cancer. *Lancet*, **ii**, 1095.

BISWAS, R. & VONDERHAAR, B.K. (1987). Role of serum in the prolactin responsiveness of MCF-7 human breast cancer cells in long-term tissue culture. *Cancer Res.*, **47**, 3509.

BONNETERRE, J., PEYRAT, J.P., BEUSCART, R. & DEMAILLE, A. (1986). Correlation between prolactin receptors (PRLR), estradiol (ER) and progesterone receptors (PgR) in human breast cancer. *Eur. J. Cancer Clin. Oncol.*, **22**, 1331.

BONNETERRE, J., PEYRAT, J.P., BEUSCART, R., LEFEBVRE, J. & DEMAILLE, A. (1987). Prognostic significance of prolactin receptors in human breast cancer. *Cancer Res.*, **47**, 4724.

COSTLOW, M.E., BUSCHOW, R.A., RICHERT, N.J. & MCGUIRE, W.L. (1975). Prolactin and estrogen binding in transplantable hormone dependent and autonomous rat mammary carcinoma. *Cancer Res.*, **35**, 970.

COX, D.R. (1972). Regression models and life tables. *J. R. Stat. Soc. B*, **34**, 187.

DAO, T.L., SINHA, D.K., NEMOTO, T. & PATEL, J. (1982). Effect of estrogen and progesterone on cellular replication of human breast tumors. *Cancer Res.*, **42**, 359.

DE SOMBRE, E.R., KLEDZIK, G., MARSHALL, S. & MEITES, J. (1976). Estrogen and prolactin receptor concentrations in rat mammary tumors and response to endocrine ablation. *Cancer Res.*, **36**, 354.

EARLY BREAST CANCER TRIALISTS' COLLABORATIVE GROUP (1988). Effects of adjuvant Tamoxifen and of cytotoxic therapy on mortality in early breast cancer. *N. Engl. J. Med.*, **319**, 1681.

FRANTZ, W.L. & TURKINGTON, R.W. (1972). Formation of biologically active $^{125}$I prolactin by enzymatic radioiodination. *Endocrinology*, **91**, 1545.

HARTREE, E.F. (1972). Determination of protein: a modification of Lowry method that gives a linear photometric response. *Anal. Biochem.*, **48**, 422.

HOLDAWAY, I.M. & FRIESEN, H.G. (1977). Hormone binding by human mammary carcinoma. *Cancer Res.*, **37**, 1946.

KAPLAN, E.L. & MEIER, P. (1958). Nonparametric estimation from incomplete observation. *J. Am. Stat. Assoc.*, **53**, 457.

KELLY, P.A., LABRIE, F. & ASSELIN, J. (1978). The role of prolactin in tumor development. In *Influences of Hormones in Tumor Development*, Vol. 2, Kellen, J.A. & Hilf, R. (eds) p. 157. CRC Press: Boca Raton, FL.

KELLY, P.A., POSNER, B.I. & FRIESEN, H.G. (1975). Effects of hypophysectomy, ovariectomy and cycloheximide on specific binding sites for lactogenic hormones in rat liver. *Endocrinology*, **97**, 1408.

LIPPMANN, M.E. (1981). Hormonal regulation of human breast cancer cells in vitro. In *Hormones and Breast Cancer: Banbury Report no. 8*, Pick, M.C., Siiteri, P.K. & Welsch, C.W. (eds) p. 171. Cold Spring Harbor Laboratory Press.

LIPPMAN, M., BOLAN, G. & HUFF, K. (1976). The effects of estrogens and antiestrogens on hormone-responsive human breast cancer in long-term tissue culture. *Cancer Res.*, **36**, 4595.

MALARKEY, W.B., KENNEDY, M., ALLRED, L.E. & MILO, G. (1983). Physiological concentrations of prolactin can promote the growth of human breast tumor cells in culture. *J. Clin. Endocrinol. Metab.*, **56**, 673.

MANNI, A., WRIGHT, C., DAVIS, G., GLENN, J., JOEHL, R. & FEIL, P. (1986). Promotion by prolactin of the growth of human breast neoplasms cultured in vitro in the soft agar clonogenic assay. *Cancer Res.*, **46**, 1669.

MANNI, A., TRUJILLO, J.E. & PEARSON, O.H. (1977). Predominant role of prolactin in stimulating the growth of 7-12-dimethylbenz(a)anthracene-induced rat mammary tumor. *Cancer Res.*, **37**, 1216.

MANTEL, N. (1966). Evaluation of survival data and two new rank order statistics arising in its consideration. *Cancer Chem. Rep.*, **50**, 163.

MCGUIRE, W.L., DE LA GARZA, M. & CHAMNESS, G.C. (1977). Evaluation of estrogen receptor assays in human breast cancer tissue. *Cancer Res.*, **37**, 637.

MURPHY, L.J., MURPHY, L.C., VRHOVSEK, E., SUTHERLAND, R.L. & LAZARUS, L. (1984). Correlation of lactogenic receptor concentration in human breast cancer with estrogen receptor concentration. *Cancer Res.*, **44**, 1963.

NAGASAWA, H. (1979). Prolactin and human breast cancer: a review. *Eur. J. Cancer*, **15**, 267.

PEARSON, O.H., MANNI, A., CHAMBERS, M., BRODKEY, J. & MARSHALL, J.S. (1978). Role of pituitary hormones in the growth of human breast cancer. *Cancer Res.*, **38**, 4323.

PICHON, M.F. & MILGROM, E. (1977). Characterization and assay of progesterone receptor in human mammary carcinoma. *Cancer Res.*, **37**, 464.

RAE-VENTER, B., NEMOTO, T., SCHNEIDER, S.L. & DAO, T.L. (1981). Prolactin binding by human mammary carcinoma: relationship to estrogen receptor protein concentration and patient age. *Breast Cancer Res. Treat.*, **1**, 233.

SCAMBIA, G., PANICI, P.B., BAIOCCHI, G., PERRONE, L., IACOBELLI, S. & MANCUSO, S. (1988). Antiproliferative effects of somatostatin and the somatostatin analog SMS 201-995 on three human breast cancer cell lines. *J. Cancer Res. Clin. Oncol.*, **114**, 306.

SETYONO-HAN, B., HENKELMAN, M.S., FOEKENS, J.A. & KLIJN, J.G.M. (1987). Direct inhibitory effects of somatostatin (analogues) on the growth of human breast cancer cells. *Cancer Res.*, **47**, 1566.

SHIU, R.P.C., KELLY, P.A. & FRIESEN, H.G. (1973). Radioreceptor assay for prolactin and other lactogenic hormones. *Science*, **180**, 968.

SHIU, R.P.C., MURPHY, L.C., TSUYUKI, D., MYAL, Y., LEE-WING, M. & IWASIOW, B. (1987). Biological actions of prolactin in human breast cancer. *Rec. Prog. Hormone Res.*, **43**, 277.

THORPE, S.M. & DAEHNFELDT, J.L. (1980). Specific binding of prolactin in human mammary tumors. *Eur. J. Cancer*, suppl.1, 45.

TURCOT-LEMAY, L. & KELLY, P.A. (1982). Prolactin receptors in human breast tumors. *J. Natl Cancer Inst.*, **68**, 381.

WASEDA, N., KATO, Y., IMURA, H. & KURATA, M. (1985). Prognostic value of estrogen and prolactin receptor analysis in human breast cancer. *Jpn. J. Cancer Res. (Gann)*, **76**, 517.

WELSCH, C.W. (1985). Host factors affecting the growth of carcinogen-induced rat mammary carcinomas: a review and tribute to Charles Brenton Huggins. *Cancer Res.*, **45**, 3415.

WELSCH, C.W. & GRIBLER, C. (1973). Prophylaxis of spontaneously developing mammary carcinoma in C3h/HeJ female mice by suppression of prolactin. *Cancer Res.*, **33**, 2939.